# Nutritional value and *in situ* degradability of oak wood roughage and its feeding effects on growth performance and behavior of Hanwoo steers during the early fattening period

**DOI:** 10.5713/ajas.19.0658

**Published:** 2019-12-24

**Authors:** Ye Ri Ju, Youl Chang Baek, Sun Sik Jang, Young Kyoon Oh, Sung Suk Lee, Yong Sik Kim, Keun Kyu Park

**Affiliations:** 1 Cargill Agri Purina, Inc. Gunsan Plant, Gunsan 54007, Korea; 2 National Institute of Animal Science, Rural Development Administration, Wanju55365, Korea; 3 Division of Wood Chemistry, Department of Forest Products, National Institute of Forest Science, Seoul 02455, Korea; 4 Division of Forest Material Science and Engineering, Kangwon National University, Chuncheon 24341, Korea; 5 Department of Animal Science and Technology, Konkuk University, Seoul 05029, Korea

**Keywords:** Oak Roughage, Steam Digestion, Animal Performance, Animal Behavior, Rumen Degradability

## Abstract

**Objective:**

This study was conducted to evaluate changes in nutritional value and *in situ* dry matter (DM) degradability of oak and pine wood before and after steam-digestion process (60 min/160°C/6 atm) and feeding effect of the oak roughage on performance and behavior of Hanwoo steers.

**Methods:**

Chemical composition and tannin concentration were analyzed for oak and pine trees before and after the pretreatment. *In situ* DM and effective degradability of these samples were assessed using a nylon bag method. *In vivo* trial was performed to estimate animal performance and behavior, using steers fed total mixed ration (TMR) diets containing 0% (control), 25% (OR-25), and 50% (OR-50) of the oak roughage. Eighteen steers were allocated into nine pens (2 steers/pen, 3 pens/treatment) for 52 days according to body weight (BW) and age.

**Results:**

By the steam-digestion treatment, the neutral detergent-insoluble fiber was decreased from 86.5% to 71.5% for oak and from 92.4% to 80.5% for pine, thereby increasing non-fiber carbohydrate. *In situ* DM degradability of treated oak reached 38% at 72 h, whereas that of untreated oak was only 11.9%. The 0 h degradability of the treated pine increased from 5.9% to 12.1%, but the degradability was unchanged thereafter. Animal performance including BW, average daily gain, DM intake, and feed conversion ratio was not different among control and oak treatments. No differences were detected in animal behavior such as lying, standing, rumination, drinking, and eating, except walking. Walking was higher in control than oak treatments with numerically higher eating and lower lying times, probably due to bulkier characteristics of rice straw in the diet.

**Conclusion:**

This study demonstrates that the oak roughage can be substituted for 50% of total forage or 100% of rice straw in TMR diets at early fattening stage of Hanwoo steers.

## INTRODUCTION

In Korea, approximately 70% of the land is mountainous and is not suitable for producing forages due to concentrated precipitation along with high temperature during the summer and very cold weather in winter. The proportion of rice straw in domestic forage is 45%, while the supply of rice straw is expected to decrease due to continuous decline in rice demand and increase in rice productivity [1]. Therefore, it is necessary to search and develop roughage substitutes when the price of imported forage increases, or the supply of rice straw is insufficient. Vast amount of thinning woods is produced annually through the Forest Management Project, estimating approximately 1.3 million m^3^ [2]. Thinned woods can promote the spread of forest fires when left in mountain areas. When used as timber, it is composed of immature material, and their physical and mechanical properties are lower than those of mature wood. Therefore, it is used primarily as fuel [3].

Various efforts have been made to use wood products as a roughage source for ruminant diet. However, lignocellulosic biomass such as wood products is difficult to decompose in the rumen without special processing. More efficient utilization of woody materials could be realized by pretreatments using steam, chemicals, mechanical milling and combinations of these treatments. Researches have been mainly conducted for bioethanol production to increase the yield of fermentable products by using heat treatment in combination with chemical treatment [4]. However, the products are not environmentally friendly, must be neutralized prior to feeding and may result in a decrease in available nutrients due to excessive solubilization of hemicellulose.

Hydrothermolysis using high pressure and temperature (150°C to 180°C is known to have a significant effect on wood and agricultural residues [4]. Previous results [5] suggest that the thinning woods from oak and pine forests treated with the steam-digestion process may be used as roughage sources by comparing chemical composition and *in situ* degradability. However, there are no published data on changes in chemical composition before and after pretreatment and the use of the treated wood products in the diets of beef cattle. Therefore, the purposes of this study were to i) investigate changes in nutritional value of oak and pine trees before and after pretreatment, ii) compare *in situ* degradability of these substrates, and iii) evaluate animal behaviors and the possibility of oak roughage after pretreatment as a substitute for rice straw of total mixed ration (TMR) for Hanwoo steers at early fattening period.

## MATERIALS AND METHODS

### Animal care

The animal use and protocols employed during the research were reviewed and approved by the Institutional Animal Care and Use Committee at Konkuk University (Approval number: KU17094).

### Exp. 1. Chemical analysis and *in situ* dry matter degradability

#### Preparation of wood-based roughages

Trees that were thinned through the Forest Management Project of the Fifth National Forest Plan (2008–2017) by Korea Forest Service [2]. Wood chips from Mongolian oak (*Quercus mongolica*) which is the major broad-leaved tree and the chips from pitch pine (*Pinus rigida*) which is a typical conifer species since the 1970s in Korea were prepared for steam-milling process.

**Figure 1:**
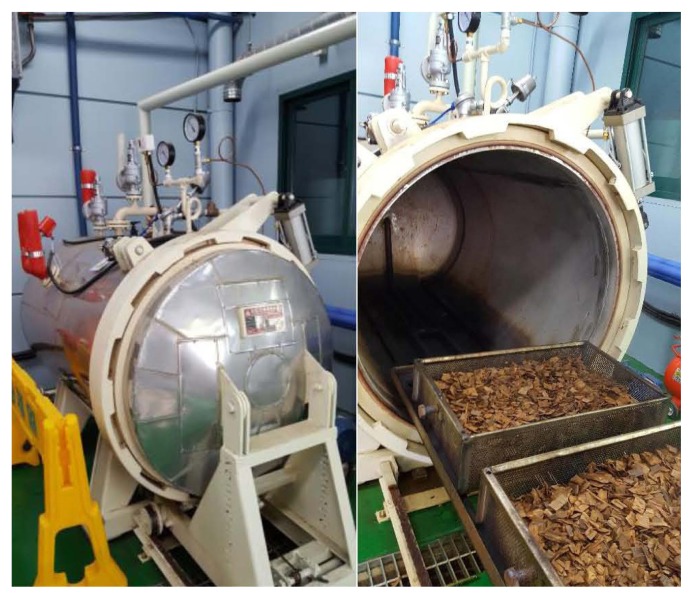
Steam digester.

**Figure 2:**
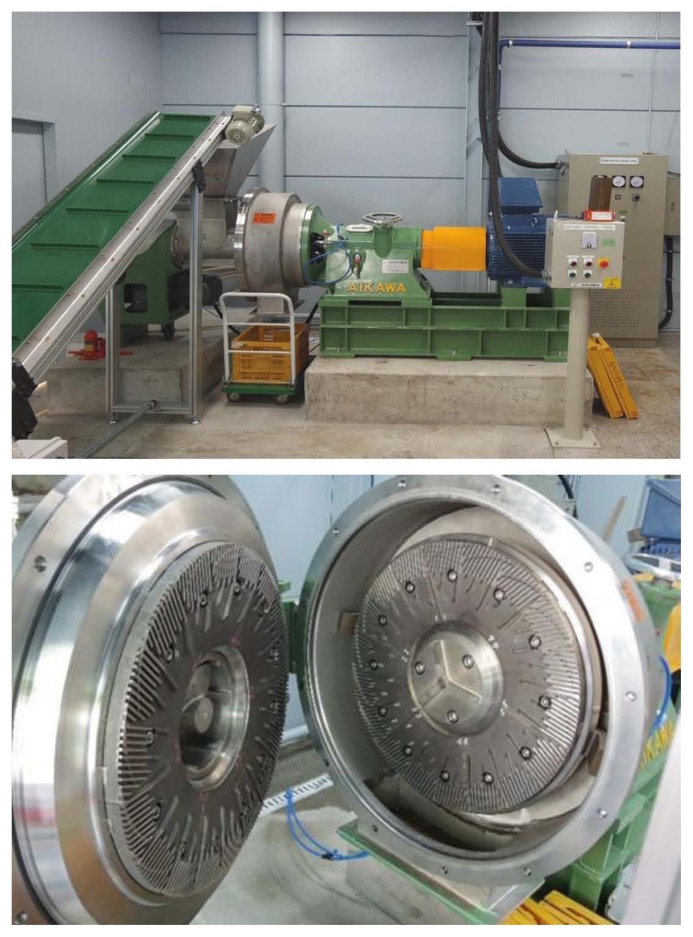
Disc mill.

The thinned wood except for leaves, branches and bark was broken into chips of 3 to 5 cm size and immersed in water for at least 2 hours before steam-milling process. The steam-milling process was conducted by the method described by Kim et al [5]. Specifically, wood chips were steamed for 60 minutes at 160°C under 6 atm. pressure, using a pilot scale steamer (Figure 1), then passed through a disc mill to produce woody roughages (Figure 2). The disk interval was 3 to 4 mm with 1,995 rpm rotation speed. Woody roughages were air-dried after processing for long-term storage.

#### Chemical analysis

Wood samples before and after steam-milling process were dried at a forced-air oven for over 24 hours at 105°C and ground through 2 mm screen using a Wiley mill (Model 4, Thomas Scientific, Swedesboro, NJ, USA). Samples were analyzed for dry matter (DM) [6] (AOAC official method 930.15), crude protein (CP) [6] (AOAC official methods 930.15 and 990.03), fat [7] (AOAC official methods 930.15 and 2003.05), lignin [8], acid detergent-insoluble fiber (ADF) [6] (AOAC official methods 930.15 and 973.18), neutral detergent-insoluble fiber (NDF) [9], and ash [6] (AOAC official method 942.05). Total tannin was measured by the Folin-Denis method [10] to test the level that could be toxic to ruminants.

#### In situ dry matter degradability

Three Holstein steers (381± 14.5 kg) fitted with ruminal cannula were fed 4 kg of tall fescue and 2 kg of a formulated concentrate mix, divided into equal portions and offered daily at 09:00 and 18:00. The steers had free access to water. Nylon bags (10×20 cm and 50 μm of pore size; Ankom Technology, Macedon, NY, USA) were filled with approximately 4 g of sample, considering the proportion of the sample weight to the bag surface area was 10 to 20 mg/cm^2^, which satisfies the optimal proportion recommended by Nocek [11]. Duplicate bags were prepared in each steer for each incubation time.

At three hours after feeding, that is expected to have sufficiently high for rumen microbial activity to degrade low digestible woody materials, the bags containing test samples were incubated in the ruminal ventral sac for 0, 4, 8, 12, 24, 48, 72, 96, and 120 h. The time of entry into the rumen for each incubation time was adjusted to minimize the number of nylon bags staying in a rumen, instead of putting all nylon bags at once and taking them out for each incubation time [12] or *vice versa* [11]. Thus, maximum number of bags in the rumen at any one time was less than 15. Upon removal, bags were washed in cold, running tap water until the rinsed water became clear, and were dried at 65°C in a drying oven for 48 h.

The degradability of DM from nylon bag fitted to the exponential model and effective degradability (ED) were calculated as follows [13];

$$\begin{array}{l} \text{P}=\text{a}+\text{b}(1-{\text{e}}^{-\text{ct}})\\ \text{ED}=\text{a}+\text{b}\times \text{c}/(\text{c}+\text{k}) \end{array}$$

Where, P is the DM degradability at time t, ‘a’ rapidly degradable fraction, ‘b’ the slowly degradable fraction, ‘c’ constant for b fraction, ‘t’ rumen suspension time and ‘k’ outflow rates in rumen, assuming an outflow rate (k) of 0.02 per hour.

### Exp. 2. Effects of dietary woody roughages on performance of Hanwoo steers

#### Experimental design

After an adaptation period of 2 weeks, eighteen Hanwoo steers of 15.5±2.5 months age (initial body weight [BW] 425±105 kg) were allocated into three experimental groups according to BW and age for 52 days (initial fattening period); i) control (TMR containing 0% of oak roughage), ii) OR-25 (TMR containing 25% of oak roughage, as fed basis), and iii) OR-50 (TMR containing 50% of oak roughage, as fed basis). Thus, the substitution level of oak roughage was 0%, 25%, and 50% for total roughage in TMR diets containing rice straw and alfalfa, being equivalent to 0%, 50%, and 100% for rice straw. Steers were housed in sawdust bedding pens (2 steers/pen; total 9 pens; 10.0 m wide×15.0 m length) and were marked with numbered tag into the ear.

The TMR diets were formulated based on the Korean Feeding Standard for growing Hanwoo steers [14] to meet energy and protein requirements (total digestible nutrient [TDN] 71% to 72%; CP 12% to 13%). The formulation and chemical composition of experimental diets for each treatment are shown in Table 1. When rice straw was replaced with oak roughage, the substitution level did not appear to be 50% for OR-25 and 100% for OR-50, because the NDF contents of these roughage sources were different but the TMR diets were formulated on an equivalent NDF basis. In addition, although the CP content of OR was very low (0.81%) compared to rice straw (5.07%), the replacement level of these roughage sources in OR-25 and OR-50 was only 3.12% and 6.24% (as fed basis), respectively, indicating a very low contribution to total CP content. Thus, the CP contents of control, OR-25, and OR-50 were 13.8%, 14.3%, and 14.6%, respectively and were not different among treatments.

Feeds were offered equally at 06:00 and 17:00 on daily basis. They could access fresh water and mineral block without any restriction during the whole experimental period. Steers were weighed at 12, 31, and 52 days before morning feeding of the whole experimental period. The DM intake (DMI) was measured every week for calculating feed conversion ratio (FCR; feed/gain). In addition, TMR diets fed were measured for *in situ* DM degradability. The method was carried out in the same manner as mentioned above.

During the middle of the feeding trial, animal behavior was observed by installing two CCTV cameras (DS-2CE16DOT-IRP, Hikvision, Hangzhou, China) in front of and behind each pen without blind spots. Individual animal was numbered with a marker at the back to facilitate the identification. Observations were performed two consecutive days from 06:00 to 20:00 (14 h = 840 min) during the daytime.

Observations were 6 kinds of behavior, including lying, standing, rumination, drinking, eating, and walking (min). Lying includes rumination with both abdomen and chest touching the floor. Standing includes rumination, drinking, eating, and walking with all four feet standing on the ground. Thus, rumination can be included in both lying and standing. Walking defined as an animal has taken 3 steps in a progressive direction, and this behavior ends when the progressive movement stops.

#### Calculations and statistical analysis

The nitrogen-free extract (NFE), non-fiber carbohydrate (NFC), TDN, and FCR values of experimental feeds were calculated as follows: i) NFE = % DM−(% ether extract [EE]+% CP+% ash+% crude fiber). ii) NFC calculated by difference: 100−[CP+(NDF− neutral detergent insoluble CP)+EE+ash] [9]. iii) TDN = 0.93×CP+0.92×(1+EE−ash−CP−NDF)+0.75×(NDF−acid detergent lignin [ADL])×(1−ADL^2/3^/(NDF)^2/3^) [15]. iv) FCR = DMI (kg)/gain (kg).

Data obtained from chemical composition before and after steam digestion-milling process of wood chips, *in situ* DM degradability and *in vivo* trial (BW, average daily gain [ADG], DMI, and FCR) were subjected to statistical analysis using the SAS PROC MIXED (SAS Inst. Inc., Cary, NC, USA). All data were analyzed by analysis of variance and PDIFF option for multiple range tests were used to determine significant differences (p<0.05) among treatments within each classification.

## RESULTS AND DISCUSSION

### Exp. 1. Chemical analysis and *in situ* dry matter degradability of wood samples before and after steam-milling process

#### Chemical analysis

The chemical composition and tannin contents of oak and pine trees before and after steam digestion process are presented in Table 2. The CP contents of untreated oak (U-O), treated oak (T-O), untreated pine (U-P), treated pine (T-P) were very low, containing 0.93%, 0.81%, 0.51%, and 0.46%, respectively, which were lower than that of rice straw (5.07%). Low concentrations of ash were also noted for U-O, T-O, U-P, and T-P (0.91%, 0.76%, 0.36%, and 0.36%, respectively) compared to rice straw (16.7%), which is favorable in terms of intake and digestibility. The NDF values of oak were decreased from 86.5% to 71.5% by the steam digestion-milling process, and those of pine decreased from 92.4% to 80.5%. Thus, levels of hemicellulose fraction (NDF −ADF) were decreased from 23.2% to 11.0% for oak and 18.2% to 7.4% for pine.

Lignocellulosic biomass such as wood and agricultural residues requires pretreatments to make cellulose and hemicellulose more accessible to the enzymes that convert the carbohydrate polymers into fermentable sugars. There are various methods of pretreatment using steam, chemicals, mechanical milling and combinations of these treatments [4]. Hydrothermolysis using steam or hot compressed water (>160°C) is generally regarded as an effective pretreatment of agricultural and hardwood biomass, whereas softwood requires harsher conditions such as the presence of acid catalysts during the steam pretreatment [4,16]. At high temperature and pressure, saturated water itself and the acetyl groups in hemicellulose act as acids, thereby dissolving links between hemicellulose and lignin from lignocellulosic biomass [4]. For example, an increase in the solubilized xylan from steam treated eucalyptus was observed above 160°C [16]. MacAskill et al [17] also observed that hemicellulose dissolution from softwood increased with pretreatment severity from 180°C to 230°C.

The ADF and ADL values were almost unchanged in this experiment because no chemical treatment using acids or alkalis was performed. Generally, for the purpose of bioethanol production, the chemical treatment is performed along with the pretreatment process in order to increase the yield of the fermentable monomers [4]. However, these chemicals may require corrosion-resistant reactors and must be neutralized to reduce their negative impacts on ruminal fermentation as well as on the environment. Furthermore, end-product inhibitions and sugar losses during lignin separation may hinder ruminal fermentation and diminish the nutritive value of wood-based roughages [18]. Meanwhile, pine trees had very high in lignin content even with the pretreatment (27.6% vs 27.3%), which is expected to cause low digestibility in the rumen.

The NFE and NFC values before and after the pretreatment were increased 11.3% and 14.7% unit for oak, and increased 6.7% and 12.5% units for pine tree, respectively. These results indicate that parts of hemicellulose are lost from cellulose or lignin by the steam digestion, resulting in the formation of soluble sugars, which could increase available carbohydrate levels to rumen microbes.

There are limited data available about changes in nutritional value of thermally treated woods for the purpose of using as a roughage resource for ruminants. Under the similar condition with longer (90 min) treatment time, Kim et al [5] reported that NDF, ADF, ADL, and NFC values of T-O were 90.1%, 83.9%, 17.3%, and 6.50%, respectively. Compared with the results from this experiment, NDF, ADF, and ADL values were higher, whereas NFC value was lower. This is probably due to the differences in oak species used. In addition, NDF, ADF, ADL, and NFC contents of the T-P were 87.9%, 86.9%, 30.6%, and 6.32%, respectively. Although nutrient data before treatment were not provided, decomposition of pine had been rarely occurred, considering low levels of hemicellulose and NFC.

After the steam treatment, the tannin content increased from 0.87% to 1.43% for oak and 0.02% to 0.56% for pine, indicating that the total DM was reduced due to the high temperature under the steaming condition, while the condensed tannin with a stable structure remained in the wood. Condensed tannin has been known to reduce palatability and digestibility of feeds for livestock [19]. According to Yang et al [20], the total accumulation and excretion of nitrogen were not affected when tannin was added up to 2.6% (DM basis) in the feed. Conversely, other researchers reported that moderate concentrations of condensed tannin (2% to 4%, DM basis) could be beneficial to animal performance and prevent bloat by reducing rumen degradation of protein and precipitating the stable protein foam [21]. However, bioactivity of condensed tannin can be different by plant species having diverse physicochemical structures [22]. Because the level of tannin in this study was reduced to 0.09% after mixing oak roughage into the TMR diet, there was no concern of metabolic disorders and tannin at this level may even help to improve the efficiency of protein digestion of the animals.

#### In situ dry matter degradability of oak and pine

**Figure 3:**
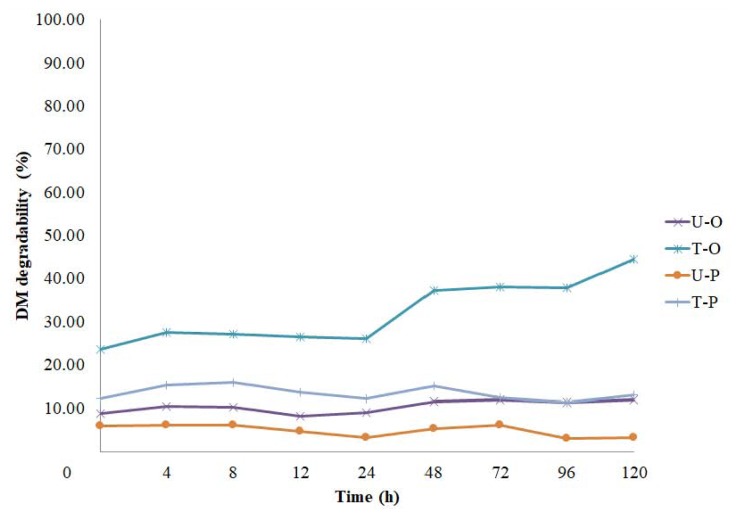
Comparison for *in situ* dry matter degradability of untreated, treated oak roughages (U-O, T-O), untreated and treated pine (U-P, T-P) by steam digestion-milling.

The *in situ* rumen DM degradability of oak and pine trees before and after steam digestion process is shown in Table 3 and Figure 3, respectively. After the steam-digestion milling process, degradability at 0 h of oak was noticeably increased (p<0.05) from 8.9% to 23.5%, mainly due to the dissolution of a portion of hemicellulose, and thereby increased NFC content. The degradability of T-O was increased after 24 h of ruminal incubation and reached to 38% at 72 h, which was close to the value (40%) of rice straw [5]. Thus, the oak roughage produced by the pretreatment could be considered as a substitute for rice straw. The difference in degradability between U-O and T-O (B-A, Table 3) exceeded 25% unit after 48 h. On the other hand, U-O had a little increase in degradability over the incubation time; the degradability at 72 h was only 11.9%.

The 0 h degradability of T-P trees was also increased from 5.9 to 12.1 (p<0.05), but the degradability of T-P almost remained the same as the incubation time progressed. Therefore, an improvement in the digestibility of pine cannot be expected through the pretreatment process in this experiment. In addition, the feeding of pine to the cattle is not generally recommended as a feed ingredient due to the side effects such as an increase in abortion rate and premature parturition due to essential oil components [23].

The steam-digestion milling process notably improved the ED of oak from 10.9% to 32.8% at outflow rate of 0.02 per h, by increasing both rapidly degradable soluble fraction ‘a’ (8.5% vs 24.8%) and insoluble slowly degradable fraction ‘b’ (3.5% vs 19.7%). On the contrary, the ED of the pine trees increased from 1.96% to 12.4% after the treatment, but this was caused by the increase in fraction a (0.77% vs 12.2%). The fraction ‘b’ was not increased at all, probably due to the very high content of lignin. These results indicate that the steam-digestion treatment could be effective altering or removing the physical and chemical impediments of oak trees, and the product can be utilized more usefully as a roughage source for ruminant livestock than pine trees.

### Exp. 2. Effects of dietary oak roughages on performance of Hanwoo steers

#### Animal performance

The BW, DMI, ADG, and FCR of control and oak treatments are shown in Table 4. Initial BW of control, OR-25 and -50 were 439.7, 454.3, and 453.0 kg, respectively. Final BW of control, OR-25 and -50 were 500.0, 521.7, and 522.8 kg, respectively, and were not statistically different among treatments. In addition, there were no statistical differences in BW among treatments during the middle of experimental periods.

The ADG of control and oak roughage treatments within each experimental period did not show any statistical difference. Thus, ADG of Hanwoo steers during the entire experimental period were not different statistically and were 1.17, 1.31, and 1.35 kg/d for control, OR-25 and -50. According to NIAS [14], the target ADG of Hanwoo steer is approximately 1.0 kg/d at the initial stage of fattening period. In this study, the ADG of all treatments during the whole experimental period exceeded 1.0 with the same level of CP and TDN in the feeding standards.

Average DMI of control and oak roughage treatments were approximately 9 kg/d (14 kg/d as fed basis) during the whole experimental period and did not show any statistical difference among treatments. This is because the amount of TMR fed was limited to the level to avoid excessive weight gain during the early fattening period, thereby preventing a decrease or rejection in feed consumption at the end of the fattening period. Prior to the animal trial, a simple palatability trial was performed by topping the oak roughage on the feeds for two weeks without mixing in a TMR, the animals were willing to consume all the feeds offered. Therefore, there was no problem in palatability for oak roughage.

During the whole experimental period, FCR of control, OR-25 and -50 were 7.90, 6.85, and 6.75, respectively, and were not statistically different among treatments. The control showed numerically higher FCR value compared to oak treatments, mainly due to slightly lower ADG. The NDF component is most critical for maintaining rumen function and health [24]. Although roughage sources have often been exchanged on an equal DM inclusion basis, some researchers suggest that substituting roughage sources on an equal NDF basis may be more appropriate [25,26]. Thus, different forage sources at a similar NDF inclusion do not impact growth performance. For example, growth performance was not influenced by forage sources such as alfalfa hay, sudangrass hay, and ground rice straw [25], and alfalfa hay, corn silage, wheat straw and corn stover [27] when fed at a similar NDF inclusion level. In this study, TMR diets were formulated at the same level of NDF as well as DM among treatments.

It is difficult to make a direct comparison with the results from other researchers because this study is the only experiment that fed oak roughage subjected to the steam-digestion process to cattle. There is also very little data available about the experiments that fed oak wood to cattle without pretreatment, probably due to low digestibility and the risk of tannin toxicity. El-Sabban et al [28] compared performance and carcass characteristics of steers fed 5% ground timothy hay and either 5% or 15% fine (FS; modulus of fineness 2.66) and coarse sawdust (CS; modulus of fineness 3.14). The ADG was higher in CS than FS treatments (5% FS, 15% FS, 5% CS, and 15% CS; 1.11 and 1.18 vs 1.22 and 1.22 kg/d, respectively), but lower than timothy-fed group (1.45 kg/d). When lambs were offered either cottonseed hulls or a ground woody product consisting of four different species of coniferous trees, BW and FCR were not affected but DMI was reduced and ADG was less [22].

**Figure 4:**
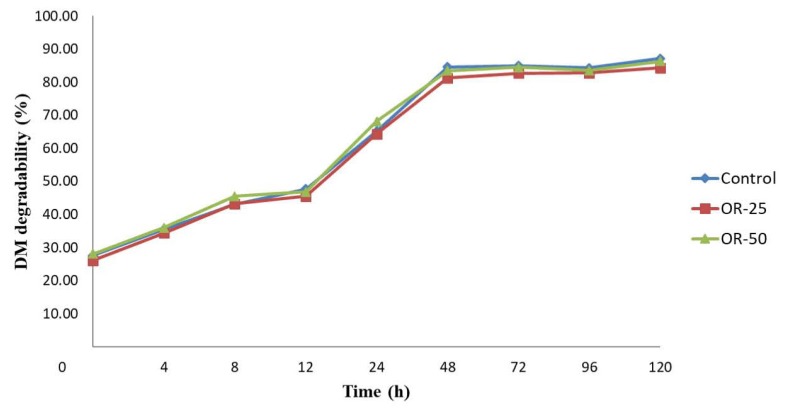
*In situ* dry matter degradability of total mixed ration diets containing oak roughage. The OR-25 and -50 represent 25% and 50% of oak roughage of total roughage in total mixed ration, respectively.

The *in situ* rumen DM degradability of the TMR diets fed to the experimental animals is presented in Figure 4. The degradation rates of control, OR-25 and -50 were 27.0%, 26.1%, and 28.1% at 0 h, then steadily increased to 46.7%, 45.5%, and 46.8% until 12 h, respectively. The degradability of control, OR-25 and -50 increased exponentially up to 84.6%, 81.2%, and 83.5% from 12 h to 48 h, respectively, then reached plateau thereafter. Lower degradability was noticed (p<0.05) for OR-25 compared to other treatments at 48, 72, and 96 h but the differences were not large. Overall, the degree and pattern of degradability were very similar among treatments because the chemical composition was formulated at the same level and feed ingredients of the TMR diets were similar except for oak roughage instead of rice straw. As previously mentioned, the *in situ* degradability of oak was close to that of rice straw. Thus, the steam-digestion process plays a role in dissolving the parts of fiber in oak, giving it a nutritional value comparable to that of rice straw.

#### Animal behavior

Table 5 presents results of animal behavior (min) after being fed TMR diets containing oak roughage. Lying behavior of control, OR-25 and -50 for 14 h were 348, 381, and 373 min, respectively, and were not statistically different among treatments during the total day time (min/14 h), time per h (min/h), and the percentage of individual behavior among total activities. Total standing includes rumination, drinking, eating and walking activities, but standing time including rumination alone is also presented to exclude the walking effect where the only significant difference was found. Nevertheless, both total standing and standing times also showed no differences among treatments.

The proportion of lying of the total behavior was 41.4%, 45.4%, and 44.4% in control and oak treatment groups, respectively. Many studies have shown that lying time decreases in warmer ambient conditions and *vice versa* [29–31]. Specifically, both time and bout frequency of standing behavior were increased when the temperature-humidity index (THI) is above 68 [30], to maximize effective surface area for heat loss from the body surfaces and(or) increase the efficiency of respiration [29,31] reported that daytime standing times (13 h) of Hanwoo steers were the longest in the summer at all growth stages (summer vs winter; growing stage 750 vs 461 min, early-fattening stage 660 vs 469 min, late-fattening stage 614 vs 483 min), and steers were found to have more variable behavioral patterns during summer. In this study, the feeding trial was conducted during early summer (from May to July), having ambient temperature ranged from 12.9°C to 31.5°C and averaged 21.4°C with THI 68.6. According to Kim et al [31], the percentage of lying of Hanwoo steers at early-fattening stage was 15.4% in summer compared to 41.7%, 32.9%, and 40.0% in spring, autumn and winter, respectively. Therefore, the proportion of lying in this study exceeded the value of spring season, and THI was not high enough to affect standing behavior.

Rumination, drinking and eating did not show any significant difference among treatments. Rumination and eating were the most common active behaviors and, as observed in other studies [31,32], time spending rumination was less compared to eating regardless of the treatment group. Pavlenko et al [32] showed that when dairy cows were transferred to a new and unfamiliar housing, they performed more rumination and walking and less eating during the first 3 d, and then it was reversed afterwards. More importantly, forage NDF source at a similar level did not impact growth performance but both forage source and inclusion level influenced feeding behavior as cattle consuming bulkier forages (wheat straw or corn stover) or at greater inclusion levels had a higher eating time [26]. Similarly, steers fed high concentrate diets containing wheat straw spent more time eating than steers fed the diets containing alfalfa with equal inclusion of NDF [33]. In addition, rumination was greatest for steers (307 min/d) consuming 10% short-grind corn stalk, followed by steers (289 min/d) consuming 5% long-grind, and was lowest for steers (245 min/d) consuming 5% short-grind [34]. These authors suggest that increasing particle size of roughage may be a means to decrease roughage inclusion rate while maintaining rumination and performance. In this study, eating time was numerically higher in control (198 min) than those of OR-25 (170 min) and OR-50 (166 min), possibly due to the bulkier nature of rice straw fed to control compare with oak treatments.

Walking activity was lower (p<0.05) in OR-25 (46.7 min/14 h) than control (102.6 min/14 h) and tended to be lower than OR-50 (70.0 min/14 h). The same patterns were also noted in time per h (min/h) and the percentage of walking of total behavior, which is reasonable because these values were derived from arithmetic ways to facilitate numerical comparisons with other studies and future researches. Consequently, the control showed numerically shorter lying time and longer eating time than oak treatments.

## CONCLUSION

Nutritional values and *in situ* degradability of oak by the steam-digestion process were improved substantially. No differences were detected among treatments in animal performance and behavior except walking. Therefore, oak roughage can be substituted for 50% of total forage or 100% of rice straw in TMR diets at early fattening stage of Hanwoo steers. In addition, although additional experiments would be required, total forage of the later stages of finishing diet could be fully replaced by the oak roughage because a relatively low amount of rice straw (10% to 15% of the total diet, DM basis) as single forage source is generally required at that time. This will contribute to the reduction of wasted forest resources and the substitution of insufficient forage sources by raising the utilization rate and added value of thinning woods, thereby reducing the production cost of farm and improving sustainability of animal husbandry. Moreover, it may be useful when there is a shortage of conventional roughage sources or when the prices soar.

## Figures and Tables

**Table 1:** Ingredients and chemical composition of total mixed ration

Items (%)	Control1)	OR-25	OR-50
Rice straw	8.45	4.23	-
Oak roughage	-	3.12	6.24
Alfalfa	6.65	6.65	6.65
Corn gluten feed	10.99	10.99	10.99
Beet pulp	7.02	7.02	7.02
Corn flake	21.27	21.27	21.27
Brewers grain TMR2)	34.98	34.98	34.98
Water	8.83	9.93	11.04
Vitamin, mineral premix	0.02	0.02	0.02
Limestone	0.65	0.65	0.65
Salt	0.63	0.63	0.63
Sodium bicarbonate	0.51	0.51	0.51
Total	100	100	100
Chemical composition (DM basis, %)			
DM	64.60	63.20	64.30
CP	13.82	14.25	14.57
EE	3.60	4.02	3.92
CF	16.29	17.50	20.21
Ash	7.15	7.41	7.33
NDF	50.88	48.22	49.77
ADF	28.02	25.77	29.23
ADL	3.45	3.46	4.73
NFE	23.74	20.02	18.27
NFC	24.55	26.10	24.41
TDN3)	71.73	72.44	69.97

TMR, total mixed rations (as fed basis); DM, dry matter; CP, crude protein; EE, ether extract; CF, crude fiber; NDF, neutral detergent-insoluble fiber; ADF, acid detergent-insoluble fiber; ADL, acid detergent lignin; NFE, nitrogen free extract; NFC, non-fiber carbohydrate; TDN, total digestible nutrients.

1) Control, 0% of oak roughage of total roughage in TMR; OR-25, 25% of oak roughage of total roughage in TMR; OR-50, 50% of oak roughage of total roughage in TMR.

2) Molasses, 8.09%; soybean hulls, 8.09%; wheat bran, 13.53%; brewers grain, 13.53%; soybean curd cake, 48.37%; rice bran, 8.09%.

3) Calculated by 0.93×CP+0.92×(1+EE−ash−CP−NDF)+0.75×(NDF−ADL)×(1−(ADL)^2/3^/(NDF)^2/3^) [15].

**Table 2:** The chemical composition of oak and pine trees before and after steam digestion-milling and rice straw (DM basis, %)

Item	U-O1)	T-O	U-P	T-P	RS
DM	91.87	94.80	93.13	94.70	90.91
CP	0.93	0.81	0.51	0.46	5.07
EE	0.11	0.71	3.60	3.00	1.99
CF	65.86	57.13	77.29	72.77	32.04
Ash	0.91	0.76	0.36	0.36	16.73
NDF	86.54	71.54	92.37	80.52	66.70
ADF	63.36	60.54	74.13	73.16	45.13
ADL	12.97	12.70	27.58	27.29	5.23
NFE	24.05	35.39	11.37	18.11	35.08
NFC	11.50	26.18	3.16	15.65	9.51
TDN2)	51.27	56.34	36.89	40.87	54.78
Total tannin	0.87	1.43	0.02	0.56	-

DM, dry matter; CP, crude protein; EE, ether extract; CF, crude fiber; NDF, neutral detergent-insoluble fiber; ADF, acid detergent-insoluble fiber; ADL, acid detergent lignin; NFE, nitrogen free extract; NFC, non-fiber carbohydrate; TDN, total digestible nutrients.

1) U-O, untreated oak (*Quercus mongolica*); T-O, treated oak; U-P, untreated pine (*Pinus rigida*); T-P, treated pine; RS, rice straw, adopted from RDA [35].

2) Calculated by 0.93×CP+0.92×(1+EE−ash−CP−NDF)+0.75×(NDF−ADL)×(1−(ADL)^2/3^/(NDF)^2/3^) [15].

**Table 3:** *In situ* DM degradability and ED of oak and pine trees before and after steam digestion-milling (%)

Time (h)	U-O1) (*A*)	T-O (*B*)	U-P (*C*)	T-P (*D*)	(*B*−*A*)	(*D*−*C*)	SEM
0	8.88^c^	23.53^a^	5.86^d^	12.10^b^	14.65	6.24	0.258
4	10.43^c^	27.55^a^	6.07^d^	15.21^b^	17.12	9.14	0.125
8	10.30^c^	27.15^a^	6.11^d^	15.83^b^	16.85	9.72	0.189
12	8.18^c^	26.41^a^	4.77^c^	13.58^b^	18.23	8.81	0.823
24	9.07^c^	26.05^a^	3.32^d^	12.23^b^	16.98	8.91	0.322
48	11.49^c^	37.25^a^	5.40^d^	14.97^b^	25.76	9.57	0.656
72	11.88^b^	38.12^a^	6.09^c^	12.47^b^	26.24	6.38	0.508
96	11.33^b^	37.86^a^	3.02^c^	11.41^b^	26.53	8.39	0.494
120	12.00^c^	44.48^a^	3.29^d^	12.93^b^	32.48	9.64	0.080
Degradation characteristics of DM and ED							
a2) (%)	8.50	24.77	0.77	12.21	-	-	-
b3) (%)	3.50	19.70	2.52	0.71	-	-	-
c4)/h	0.042	0.014	0.018	0.007	-	-	-
a+b5) (%)	12.00	44.48	3.29	12.93	-	-	-
ED6) (%)	10.86	32.75	1.96	12.41	-	-	-

DM, dry matter; ED, effective degradability; SEM, standard of error of means.

1) U-O, untreated oak (*Quercus mongolica*); T-O, treated oak; U-P, untreated pine (*Pinus rigida*); T-P, treated pine.

2)–5) Constants mean the equation P = a +b (1−e^−ct^); ED = a+b*[c/(c+k)] where “P” is; “a”, rapidly degradable fraction, “b”, insoluble fraction but degraded over time in rumen, “c”, constant for b fraction, “a+b”, potentially degradable fraction (Orskov and McDonald [10]).

6) Calculated with outflow rates of 2%, t = 120 h.

a–d Means within rows that do not share a letter differ at p<0.05.

**Table 4:** Effects of dietary oak roughage on performance of Hanwoo steers at initial fattening stage

Item	Treatments1)			SEM

	Control	OR-25	OR-50	
BW (kg)				
Initial (0 d)	439.7	454.3	453.0	18.729
Second (12 d)	454.0	470.7	472.8	16.709
Third (32 d)	482.3	497.2	499.2	17.275
Final (52 d)	500.0	521.7	522.8	18.506
ADG (kg/d)				
0 to 12 d	1.58	1.55	1.78	0.205
13 to 31 d	1.46	1.49	1.35	0.112
32 to 52 d	1.05	1.26	1.04	0.088
0 to 52 d	1.17	1.31	1.35	0.078
DMI (kg/d)				
0 to 12 d	8.98	8.85	9.00	0.012
13 to 31 d	9.03	8.85	9.00	0.003
32 to 52 d	9.01	8.81	8.96	0.018
0 to 52 d	9.01	8.83	8.98	0.009
FCR (F/G)				
0 to 12 d	5.97	6.08	5.32	0.778
13 to 31 d	6.30	6.37	6.71	0.486
32 to 52 d	8.86	7.02	8.80	0.655
0 to 52 d	7.90	6.85	6.75	0.440

SEM, standard error of means; BW, body weight; ADG, average daily gain; DMI, dry matter intake; FCR, feed conversion ratio.

1) Control, 0% of oak roughage of total roughage in TMR; OR-25, 25% of oak roughage of total roughage in TMR; OR-50, 50% of oak roughage of total roughage in TMR.

**Table 5:** Observation of animal behavior (min) in Hanwoo steers fed TMR diets containing oak roughage during the day time (14 h)

Item	Lying	Total standing1) (Standing)2)	Rumination3)	Drinking	Eating	Walking
Time (total min/14 h)						
Control4)	348.1	492.9 (184.6)	107.5	7.4	198.3	102.6^a^
OR-25	381.4	458.7 (231.2)	101.9	11.1	169.7	46.7^b^
OR-50	372.6	467.3 (224.1)	90.4	6.8	166.4	70.0^ab^
SEM	22.28	22.82 (16.09)	11.37	1.56	14.39	21.20
Time (min/h)						
Control4)	24.86	35.20 (13.18)	7.68	0.53	14.16	7.33^a^
OR-25	27.24	32.75 (16.51)	7.28	0.79	12.12	3.33^b^
OR-50	26.61	33.32 (15.95)	6.46	0.48	11.89	5.00^ab^
SEM	1.61	1.98 (0.86)	0.81	0.11	1.03	1.51
Percentage (%)						
Control4)	41.44	58.66 (21.97)	12.80	0.88	23.60	12.21^a^
OR-25	45.40	54.60 (27.52)	12.13	1.32	20.20	5.56^b^
OR-50	44.36	55.21 (26.27)	10.76	0.80	19.81	8.33^ab^
SEM	2.72	2.72 (2.11)	1.35	0.19	1.71	2.52

TMR, total mixed rations; SEM, standard error of means.

1) Include rumination, drinking, eating, and walking.

2) Exclude drinking, eating, and walking.

3) Included in both lying, and standing.

4) Control, 0% of oak roughage of total roughage in TMR; OR-25, 25% of oak roughage of total roughage in TMR; OR-50, 50% of oak roughage of total roughage in TMR.

a,b Means within a column in a same unit of measurement that do not share a letter differ at p<0.05.
